# Adhesive Capsulitis of the Ankle (Frozen Ankle): An Infrequent Syndrome

**DOI:** 10.3390/biomedicines11092461

**Published:** 2023-09-05

**Authors:** Daniel Aguilar-Núñez, Dina Hamed-Hamed, María Aguilar-García, María Cuevas-Cervera, José Javier Pérez-Montilla, Ana González-Muñoz, Leo Pruimboom, Santiago Navarro-Ledesma

**Affiliations:** 1 Department of Nursing and Podiatry, Faculty of Health Sciences, University of Malaga, Arquitecto Francisco Penalosa 3, Ampliación de Campus de Teatinos, 29071 Malaga, Spain; daguilarn.tic@gmail.com; 2 Department of Physiotherapy, Faculty of Health Sciences, Campus of Melilla, University of Granada, Querol Street, 5, 52004 Melilla, Spain; dhamed@correo.ugr.es (D.H.-H.); maguilar.fisioterapia@gmail.com (M.A.-G.); maaricuevass@correo.ugr.es (M.C.-C.); perezmontilla@correo.ugr.es (J.J.P.-M.); agonzalezm@correo.ugr.es (A.G.-M.); 3 Clinica Ana Gonzalez, Avenida Hernan Nuñez de Toledo 6, 29018 Malaga, Spain; 4 PNI Europe, 2518 JP The Hague, The Netherlands; leo@cpnieurope.com

**Keywords:** frozen ankle, low-grade inflammation, chronic hypoxia, fibrosis, insulin resistance, sedentary lifestyle

## Abstract

Adhesive capsulitis, characterized by progressive fibrosis, causes a gradual, painful loss of both active and passive articular motion, leading to the final contracture of the joint capsule. The condition commonly referred to as “frozen ankle” (FA), which Goldman was the first to use, relates to the ankle joint and is challenging to both diagnose and treat. Data acquired from people who suffer from this type of damage in other joints such as the shoulder, hip, and wrist also exists. Despite the fact that a well-defined model for the medical management of FA does not exist, a wide spectrum of local treatments, both surgical and non-surgical, exist. This review gives an overview of the current scientific position of the frozen ankle in terms of evolutionary factors, etiology, the different mechanisms of action involved, current treatment options, and other possible interventions based on recent discoveries of pathophysiological mechanisms. The application of extracorporeal shockwave therapy, stretching exercises, and corticosteroid injections combined with physical therapy modalities that enhance pain management, range of motion, and functional capacity is highly advisable for the treatment of adhesive capsulitis, commonly known as “frozen joints”. Furthermore, the addition of interventions both impacting and analyzing chronic hypoxia, low-grade inflammation, and sedentary life is proposed.

## 1. Introduction

Described by Putnam in 1882, adhesive capsulitis (AC) manifests as restricted joint movement and pain occurring at the extremes of motion [1]. Progressive fibrosis leads to a gradual and painful loss of both active and passive range of motion, ultimately resulting in joint capsule contracture. Individuals affected by this condition endure months to years of pain and disability [2]. AC is classified into two forms: primary and secondary [3]. The primary form is idiopathic, characterized by the gradual onset of joint stiffness and pain [4]. The secondary form, attributed to various predisposing factors, has been further categorized into intrinsic, extrinsic, and systemic factors by several review articles, depending on their nature [3,4,5]. While adhesive capsulitis of the shoulder is a commonly encountered clinical syndrome [4,6], its occurrence in other joints is rare and has only been reported in the hip joint [7] and ankle [8,9].

When adhesive capsulitis is suspected, arthrography plays a crucial role, revealing reduced synovial fluid volume (typically less than 8 mL) and increased intra-articular pressure [1]. Several risk factors associated with AC development include cancer, chronic regional pain syndrome, diabetes, Dupuytren’s syndrome, heart and neck surgery, nephrolithiasis, Parkinson’s disease, shoulder injury, smoking, and thyroid disease (particularly hypothyroidism) [10]. Thus, understanding the shared pathways among these comorbidities and the frozen ankle presents an opportunity for the development of novel treatments. For instance, low-grade inflammation (LGI) and insulin resistance, which share common pathophysiological mechanisms, hold potential as targets for innovative therapeutic approaches [11].

The condition commonly referred to as “frozen ankle” (FA), which Goldman [1] was the first to use, relates to the ankle joint and is challenging to both diagnose and treat [2]. The joint, which on clinical presentation is stiff, painful, and swollen, exhibits a reduced range of motion and may show calf muscle atrophy and swelling upon physical examination [3]. The literature infrequently reports adhesive capsulitis of the ankle (ACA). However, its occurrence is suggested to be secondary to trauma like pilon or ankle fractures or chronic ankle sprains [2], such as inversion-type injuries with all their potential complications, ranging from simple sprains to lateral ligament ruptures or fractures of the lateral malleolus.

The incidence of ACA is much lower than that of adhesive capsulitis in the shoulder, with studies suggesting it may occur with more frequency than realized [7,12]. In most cases, ACA appears as a result of repeatedly spraining or fracturing an ankle. Idiopathic ACA risk factors include infection, heart disease, autoimmune disorders, and diabetes. The list of potential risk factors is headed by diabetes, with type 1 affecting up to 40 percent of adults and type 2 affecting 10 to 36 percent, who go on to develop AC of the shoulder.

Effective management and diagnosis of ACA are difficult. Limited research on the subject was found after a thorough review of the existing literature; most studies were directed toward adhesive capsulitis of the shoulder. Thus, the aim of this study is to carry out a review of the currently available literature on “frozen ankle” and adhesive capsulitis of the ankle, which is an infrequent syndrome, to show current treatment options and novel systemic approaches. Additionally, this study aims to expand the knowledge available on less well-known risk factors and pathophysiological mechanisms that lead to the development of FA and to suggest new treatments aimed primarily at impacting low-grade inflammation (LGI) and insulin resistance.

## 2. Pathophysiology 

The common etiologies of adhesive capsulitis (ACA) are infection, trauma, and surgery. Subsequent to these events, the possible development of fibrous scar tissue in the posterior or anterior capsule, or both, predisposes the patient to increased fibrosis, although the cause and effect are still unclear [13]. The development of arthrofibrosis could be linked to prolonged immobilization after surgery or trauma, especially when the ankle is fixed in the equinus position [14]. A principal cause of arthrofibrosis in all joints is inflammation, regardless of its origin, for example, infection, systemic disease, surgery, or trauma. The scientific community is exploring the role of the inflammatory cycle more and more [15], specifically the role that cytokines such as interleukin-1 (IL-1) and interleukin-6 (IL-6) play. These cytokines often upregulate during infection and are being researched as they cause adhesion, increased matrix metalloproteinase activity, and cell migration [13].

The precise underlying mechanisms of post-traumatic adhesive capsulitis remain elusive. Nevertheless, the symptoms of a frozen ankle closely resemble those of a frozen shoulder, with the pathological processes occurring inside the tissues also appearing to be similar. The entire shoulder joint capsule has been shown to be involved following pathology studies, not only the initial site of injury. The thickening of the capsule’s fibrous layer by connective tissue, which contains new fibrocytes and is organized in dense, compact bundles, marks the first abnormality [16,17]. In these patients, dorsiflexion rather than plantarflexion is predominately lost, and as suggested by the arthrography findings in a frozen ankle, the intra-articular volume is markedly reduced and the normal recesses at the anterior and posterior compartments are lost [18].

There are key mechanisms that develop in frozen shoulder (FS), and these may also exist in ACA. These include advanced glycation end product (AGE) accumulation in the shoulder [19], which is related to a resistance to insulin and compensatory hyperinsulinemia [20], chronic hypoxia [21], chronic low-grade inflammation, and endotoxemia. A person’s physical activity level is not the only factor involved in a sedentary lifestyle since there is also an association with increased inflammatory activity and insulin resistance development [22,23]. Furthermore, an increase in the accumulation of free radicals, AGEs, and no doubt subclinical changes in the extracellular matrix and connective tissue may be caused by oxidative stress, which is associated with the presence of inflammatory cytokines [19]. On the one hand, different areas of the brain may undergo reorganization at the neuroanatomical level, which is similar to the process in phantom pain, as a result of failure to use a body part [24,25]. It is largely accepted that any neuropathic mechanism is characterized by the ectopic generation of action potentials in somatosensory afferent fibers [26] or pain production or neglect syndrome in the affected body part [27]. Both the fear of pain and the knowledge that rarely used tissue may be more easily damaged can produce a fear-based reaction in the brain that provokes a muscle defense response, which potentially leads to a frozen ankle or frozen shoulder [27,28]. The non-dominant joint is the most affected [29], and it is most commonly the left. This may be indirect evidence that part of the pathophysiology of a frozen ankle (more evidence and research) and a frozen shoulder is linked to a lack of movement and a weakening of the various joint tissues (i.e., capsule, ligaments, synovial sheath), consequent to “cerebral” joint immobilization and the muscle defense associated with it. Additionally, the fact that hemiplegia sufferers are at greater risk of suffering from frozen shoulder [20,30] is extra validation that a frozen ankle or frozen shoulder may be the result of a lack of mobility linked to muscular defense or a neglect syndrome [31]. There is significant evidence that a neglect syndrome results from having had hemiplegia, which may explain the risk of suffering a frozen shoulder after a stroke [27]. In sum, modern sedentary humans’ underuse of the full range of motion appears to result in a predisposition to the development of adhesive capsulitis syndromes such as FS or FA.

The occurrence of a frozen shoulder or frozen ankle (FA) is associated with capsulitis syndromes. Molecular biological studies indicate the presence of angiogenesis, infiltration of inflammatory cells, and elevated levels of inflammatory cytokines such as cyclooxygenase (COX)-1, COX-2, IL-1, IL-6, and tumor necrosis factor-alpha (TNF-α) in frozen shoulder. At the molecular level, this inflammation may serve as an initial manifestation of frozen shoulder, with the triggering, regulation, and subsiding of inflammation influenced by COX-1, COX-2, IL-1, IL-6, and TNF-α [28]. Chronic low-grade inflammation or infection may predispose individuals to the development of frozen shoulder [16,32].

Following a triggering event, a synovial inflammatory response is activated, leading to the proliferation of fibroblasts and significant deposition of extracellular matrix proteins [13,33,34]. This results in an increased distance between the tissue and blood vessels, impairing blood flow and reducing oxygen delivery to the injured tissue, ultimately causing local hypoxia [13,35]. Lack of joint movement contributes to chronic hypoxia in a low partial oxygen pressure (pO2) environment [36]. Consequently, this creates a suitable setting for an inflammatory process to occur, mediated by the activation of transcription factors such as hypoxia-inducible factor 1 (HIF-1) and nuclear factor Kappa B (NF-kB) [33,36,37]. Additionally, various vascular and endothelial growth factors (VEGF) and matrix metalloproteinases (MMP 1, MMP 3, and MMP 13) are activated, which are associated with angiogenesis, inflammation, and tissue destruction [36]. Oxygen deprivation triggers the release of inflammatory cytokines, particularly TNF.

**Figure 1:**
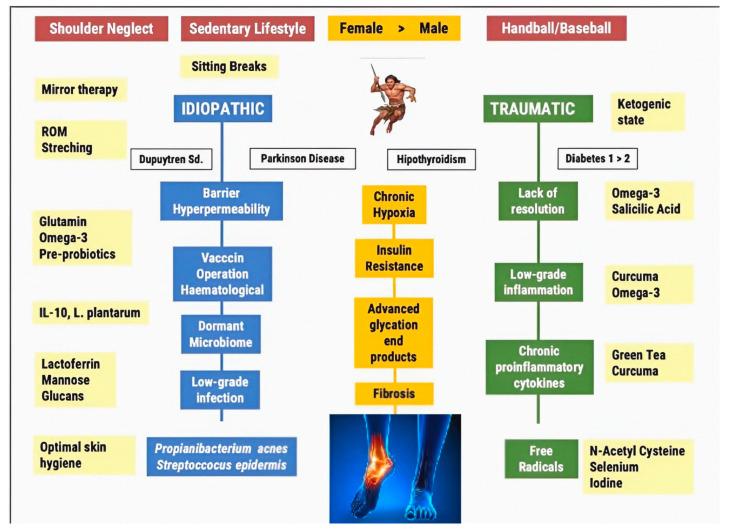
Current and proposed pathways leading to Frozen Foot Syndrome.

De la Serna et al. demonstrated the existing and suggested mechanisms responsible for the development of frozen shoulder syndrome [6]. By extrapolating these findings, a deeper and more comprehensive comprehension of frozen foot syndrome can be attained. Figure 1 presents a proposed framework outlining the pathophysiology of FA, encompassing both diagnostic and therapeutic aspects. The mechanisms associated with the “idiopathic” progression of frozen ankle are represented by the color blue in the diagram.

Green indicates the mechanism associated with the development of traumatic FA, such as repeatedly occurring throws in sports. Yellow indicates the treatments that target the action mechanisms and risk factors that cause this obscure pathology. Finally, white indicates those pathologies that are linked to a higher predisposition to developing FA.

Current knowledge about potential risk factors and underlying factors in the development of FS has advanced due to the cited data, which has led to a better understanding of how FS develops. In contrast, no reported evidence exists for FA. Although the breakthrough in FS is clear, the frozen ankle is yet to be fully understood; epidemiological data suggests that further research is needed [6], and even more so in the field of the frozen foot [6]. 

## 3. Diagnosis

Frozen ankle, which is mainly diagnosed by clinical assessment, most frequently appears as deep pain in the posterior ankle. Prior to considering arthroscopic treatment, it is important to clinically evaluate the presence of extra-articular ankle stiffness resulting from the adhesion of periarticular muscles or tendons. Additionally, if there is limited passive dorsiflexion of the toes when the ankle is in dorsiflexion, it can be indicative of tight long toe flexors. Similarly, limited passive plantar flexion of the toes when the ankle is in plantar flexion may suggest tightness in the long toe extensors [18]. 

**Figure 2:**
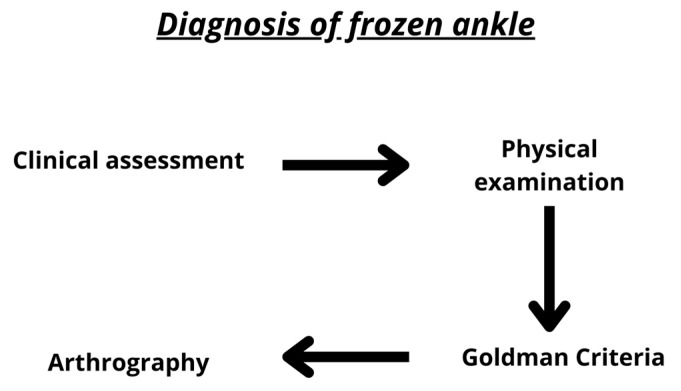
Criteria diagnosis of frozen ankle.

In the present day, ankle arthrography is considered the most effective method for diagnosing frozen ankles when necessary [16]. Magnetic resonance imaging (MRI) serves as a valuable tool for ruling out certain pathologies such as inflammation, ligament ruptures, and loose bodies. However, its diagnostic value in ankle adhesive capsulitis remains uncertain [16]. Patients experiencing ankle pain and stiffness should undergo a diagnostic arthrogram following the criteria set forth by Goldman et al. [8] (Figure 2). A positive arthrogram would then prompt an MRI of the ankle and therapeutic ankle arthroscopy [16]. It is worth noting that the criteria employed by Goldman et al. [8] for diagnosing adhesive capsulitis may have been overly restrictive, as patients often exhibit arthrofibrosis and scarring in the ankle joint, potentially not extending to the anterior recess. Implementing a straightforward diagnostic protocol that leads to more frequent reporting of this condition could result in increased detection of ankle caps. 

Physical examination, though non-specific, may reveal some muscle wasting and limitation in the normal range of active and/or passive movement; plain film findings are likewise usually non-specific [9].

Although adhesive capsulitis of the ankle seems to present in a similar way, there are currently no clinical criteria for its proper diagnosis or representation.

## 4. Treatment

The medical management of frozen shoulder has no precise model to follow; nevertheless, a broad range of surgical and non-surgical local treatments are available.

Adhesive capsulitis, or “frozen joint,” displays a general loss of active and passive movement as one of its distinctive features. In patients with stage 2 and 3 adhesive capsulitis, the use of extracorporeal shock wave therapy, exercises for stretching, and injections of corticosteroids in conjunction with physical therapy techniques and methods that improve the pain, range of motion, and functional status can be strongly recommended. The implementation of laser therapy with other conventional treatments is highly recommendable for pain relief and for improving the range of motion and functional status of patients in stage 2 [38,39].

The reported number of patients who have received treatment for ACA is significantly low, and no long-term outcome data exists. Symptoms usually improve through the use of physical therapist-supervised exercise programs that focus on the ankle’s range-of-motion; however, a lack of documented long-term benefits exists. Furthermore, the literature does not discuss the use of complementary approaches such as whirlpools, manipulation under anesthesia, ultrasound (US), and intra-articular steroid injections. The arthroscopic management of ACA has demonstrated notable advantages compared to corticosteroid injections and other non-surgical therapies [16]. A study involving three patients who underwent arthroscopic surgery reported satisfactory outcomes, with one patient being followed up for a duration of two years [16]. Furthermore, the literature highlights the effectiveness of an endoscopic approach for treating FA [39] and identifies arthroscopic synovectomy as an efficacious method for addressing posttraumatic adhesive capsulitis of the ankle [16]. Posttraumatic ankle stiffness can severely impede daily functioning, necessitating the identification of its underlying cause to establish an appropriate treatment plan (Table 1).

During the initial stages of acute adhesive capsulitis, joint movement may aid in reducing inflammation and preventing the formation of fibrous adhesions. Patients may receive anti-inflammatory medications or steroid injections while also being encouraged to engage in continuous movement to maintain the full range of motion in the joint. Ankle stiffness can be attributed to either extra-articular or intra-articular factors. Tendon adhesions or muscle contractures surrounding the ankle commonly contribute to an extra-articular posttraumatic stiff ankle. While the arthroscopic capsular release of the ankle joint is suitable for symptomatic ACA cases unresponsive to conservative treatment, it is contraindicated for ankle stiffness caused by degenerative joint disease, intra-articular malunion, or extensor adhesions of the ankle [16]. Unfortunately, routine ankle arthroscopy techniques can pose challenges when attempting arthroscopic examination of the posterior ankle compartment and posterior ankle capsulectomy.

At present, the most beneficial interventions for individuals experiencing the aforementioned conditions involve mobilization techniques for the ankle joint and physical therapy that emphasizes both active and passive range of motion [6].

Multiple techniques like exercise, electrical healing, and massage are used from the very beginning if the patient with adhesive capsulitis is being treated through physiotherapy. With the help of massages, heat therapy, cryotherapy, ultrasonic rays, transcutaneous electrical nerve stimulation (TENS), and extracorporeal shock wave therapy, only the associated pain might be reduced [39]. This may be a short-term benefit to have. These techniques improve tissue-based metabolic functions, make the tissue more flexible, bring a rise in the pain threshold, and modify neuromuscular activity, which causes the muscles to relax [39]. To treat adhesive capsulitis, one of the most frequently used methods is the administration of intra-articular corticosteroid injection [9,16]. Nowadays, fibrosis associated with adhesive capsulitis shows new perspectives for future treatment options, such as the use of stem cells, mesenchymal stromal cells, and pluripotent nonhematopoietic stem cells with self-renewal capability, which are being intensively investigated in clinical trials [26]. On the one hand, platelet-rich plasma (PRP) has grown to be the technique to treat frozen shoulders; it is believed that it stimulates the revascularization of soft tissue and enhances the growth factors’ concentration, which provides an improvement in the speedy healing of tendons [39]. On the other hand, extracellular vesicles (EVs), a heterogeneous group of cell-derived membranous structures, are involved in multiple physiological and pathological processes [26]. Recent evidence has shown that EVs play a role in many skeletal disorders, including osteoporosis, rheumatoid arthritis, and bone fracture [26]. Bursa tissue is a rich source of mesenchymal stem cells adjacent to the rotator cuff, and therefore recent studies believe that bursal cells are a good biologic augmentation to the otherwise vulnerable repair site of the torn rotator cuff in frozen shoulder [40]. Therefore, this should be further studied in both clinical and research settings when treating those suffering from FA.

Suggested Approaches Derived from Theoretical Mechanisms

Despite the circumstances, we strongly advocate for a comprehensive global study of FA. The available literature in this area is limited. Given the systemic nature of the postulated pathogenic mechanisms underlying ankle adhesive capsulitis, or FA, and considering the scarcity of publications on this approach, we propose a series of FS interventions. These interventions aim to target the described mechanisms of action and complement current treatments, which, in our opinion, deserve further investigation in the field of FA [6]. Applying the knowledge and evidence from frozen shoulder (FS) is crucial to extrapolating their findings to FA. Today’s lifestyle exposes individuals to various health risk factors [41]. Hormonal factors associated with chronic stress, as well as dietary factors like increased consumption of food additives or gliadin-rich cereals, pose threats to cell-binding complexes [6,42]. Heightened permeability of bodily barriers increases the risk of endotoxemia, chronic inflammation, and insulin resistance [6,43]. We speculate that multiple signaling pathways, suggested as mechanisms associated with FS, could be positively influenced by different compounds present in plants. One key alarmin believed to play a crucial role in perpetuating frozen shoulder is HMGB1 [44], which is released by activated macrophages/monocytes and acts as a late inflammatory mediator [45]. It binds to the Receptor for Advanced Glycation End Products (RAGE), activating mitogen-activated protein kinases and NF-kB. Recent studies have explored several herbal medicines as HMGB1 inhibitors [46]. Epigallocatechin and epicatechin, which are polyphenolic compounds found in green tea (Camellia sinensis), are among them.

The pain experienced in FS is nociceptive and arises from peripheral abnormalities associated with the capsular structures. However, chronic inflammatory mediators also contribute to the process of central sensitization [47]. Cytokines and other local inflammatory components released within the inflamed environment may be responsible for both central and peripheral sensitization [48]. In this context, mirror therapy has demonstrated effectiveness in patients with conditions such as phantom limb, complex regional pain syndrome, or stroke where central sensitization is present [40]. Mirror therapy is a simple, non-invasive, and cost-effective approach that involves using a mirror to reflect the movement of the unaffected body part while keeping the affected limb hidden from view. The rationale behind mirror therapy is based on the brain perceiving the affected arm as normal and serves as an anti-neglect intervention through visual stimulation [40].

Despite the prevailing paradigm suggesting that FA resolves on its own, further research is required to validate this notion. The systemic approach proposed in this review could serve as the missing link to enhance the success rate of individuals suffering from FA.

## 5. Discussion 

The aim of the current study is to analyze the present literature on “frozen ankle” and adhesive capsulitis of the ankle, which is an infrequent syndrome. Additionally, to expand the available knowledge on those risk factors that are less well-known and the pathophysiological pathways that are involved in the development of a frozen ankle, Furthermore, to suggest new treatments for FA that use these altered pathways and factors as their basis, we believe that FA must be studied more globally since the current literature is limited in this regard. Nevertheless, given that the suggested pathogenic pathways for ACA or FA are of an intrinsic nature and that publications that use this approach are limited, our proposal is a series of FS interventions that focus on the outlined mechanisms of action that, in our opinion, are perfectly complementary to current treatments and deserve to be researched in the field of FA [6]. It is very important for the results that are obtained and the knowledge that is applied in FS to be extrapolated to FA. Furthermore, there are many health risk factors that can be attributed to today’s modern lifestyle [41].

The integrity of cell-binding complexes can be threatened by hormonal factors, for example, those linked to long-term stress or factors of a dietary nature, which include consuming more additive-rich foods or a large amount of cereals rich in gliadin [6,42]. An elevated risk of chronic inflammation, endotoxemia, and insulin resistance is linked to an increase in the permeability of body barriers [6,43]. Different compounds that are found in plants are thought to influence multiple signaling mechanisms (allegedly related to FS). High-mobility group protein B1 (HMGB1) is considered to be one of the key alarmins for the perpetuation of FS [44]. The release of HMGB1 is attributed to activated macrophages/monocytes that function as mediators of late-stage inflammation [45]. When HMGB1 attaches to the receptor for advanced glycation end products (RAGE), it triggers the activation of NF-kB and mitogen-activated protein kinases. In recent times, multiple herbal medicines have undergone testing as inhibitors of HMGB1 [46]. Notably, epicatechin and epigallocatechin, which are polyphenolic compounds derived from green tea (Camellia sinensis), have shown promise in this regard.

The nociceptive pain experienced in frozen shoulders arises from various peripheral abnormalities associated with the capsule’s structures. However, chronic inflammatory mediators, including cytokines and other local inflammatory components released within the inflamed environment, can influence the process of central sensitization [40,48]. These mediators may contribute to both central and peripheral sensitization. Mirror therapy has shown positive outcomes in patients with conditions such as complex regional pain syndrome, phantom limb, or stroke, characterized by the presence of central sensitization [40]. This therapy involves the use of a mirror to reflect the movement of the unaffected body part while concealing the affected area. It is a cost-effective, straightforward, and non-invasive approach. The rationale behind mirror therapy lies in the brain’s ability to perceive the affected limb as normal, serving as an anti-neglect intervention through visual stimulation [40].

The frozen ankle does not exhibit spontaneous improvement, necessitating further research to validate this notion and perpetuating the existing paradigm. The suggested systemic approach in this review holds the potential to enhance the success rate of treatment for individuals suffering from frozen ankles.

Although loss of movement in the ankle joint is frequent in the clinical setting, there is a scarcity of information in the current literature, and little is still known about it. Further studies are yet to be done, specifically those analyzing chronic hypoxia and low-grade inflammation as well as modern lifestyle choices, such as a sedentary lifestyle. In this regard, the use of physiological stress as a trigger to improve mitochondrial functioning in multiple diseases in general and FA in particular, providing a comprehensive, root-cause-focused, integrative approach to recovering health, has been proposed. Clinical interventions such as nutritional (fermented food, phytomelatonin), cold and heat exposure, and breathing techniques should be further studied [49]. Additionally, more studies are needed to establish rigorous diagnostic criteria to clinically diagnose ACA, just like those in frozen shoulder. Frozen ankle is a dysfunction that may be related to pathologies such as diabetes, Dupuytren’s syndrome, hypothyroidism, and Parkinson’s disease, and hence should also be studied.

## 6. Conclusions

Despite the high prevalence of ankle joint mobility impairment in clinical practice, there remains a dearth of comprehensive literature and limited knowledge in this area. Additional investigations are required, particularly those exploring low-grade inflammation, chronic hypoxia, and the impact of contemporary lifestyles, including sedentary habits. These mechanisms indicate that the underlying disease process of ACA extends throughout the body. Given the systemic nature of this pathology, an intervention of equal scope is warranted. Consequently, it appears that ACA does not exhibit spontaneous resolution. In addition to established conventional treatments such as extracorporeal shockwave therapy, stretching regimens, and corticosteroid injections combined with physical therapy techniques, the comprehensive systemic strategy delineated in this review presents itself as a vital component to increasing the efficacy of managing individuals afflicted by ACA.

Furthermore, there is a pressing need for further studies to establish rigorous diagnostic criteria for the clinical diagnosis of adhesive ACA, akin to the approach taken with a frozen shoulder. FA represents a dysfunction that could potentially be linked to various pathologies such as diabetes, Parkinson’s disease, Dupuytren’s contracture, and hypothyroidism, warranting thorough investigation. 

## Figures and Tables

**Table 1:** More important surgical and non-surgical local treatments available for adhesive capsulitis, or “frozen joints.”

Non-Surgical Treatment	Surgical or Injections Treatment	Pluripotent Non-Hematopoietic Stem Cells THERAPY
Extracorporeal shock wave therapy	Arthroscopic	PRP
Exercises for stretching	Corticosteroid injections	Mesenchymal stromal cells
Manual physical therapy techniques	Endoscopic	
Laser therapy Ultrasound		

## Data Availability

The data is available to consult.
